# Prevalence of Glenohumeral Internal Rotation Deficit and Sex Differences in Range of Motion of Adolescent Volleyball Players: A Case-Control Study

**DOI:** 10.3390/healthcare10112263

**Published:** 2022-11-11

**Authors:** Yasuaki Mizoguchi, Kenta Suzuki, Naoki Shimada, Hiroyuki Naka, Fumihiko Kimura, Kiyokazu Akasaka

**Affiliations:** 1 Graduate School of Medicine, Saitama Medical University, 981 Kawakado, Saitama 350-0496, Japan; 2 Kimura Orthopedic Clinic, Kumagaya, Saitama 360-0811, Japan; 3 Department of Rehabilitation, Kurando Orthopedic Clinic, 33-2 Sasame-minami, Saitama 335-0035, Japan; 4 Department of Rehabilitation, Saitama Medical University Hospital, 38 Morohongo, Saitama 350-0451, Japan

**Keywords:** youth sports, volleyball, shoulder, range of motion, sex differences

## Abstract

Shoulder range of motion (ROM) adaptation is common observed among volleyball players, but studies on the shoulder joint function of adolescent athletes are lacking. This study aimed to clarify the prevalence of glenohumeral internal rotation deficit (GIRD) among adolescent players and differences in ROM based on sex. A questionnaire survey and ROM measurements of the shoulder joint and trunk using a plastic goniometer were conducted on 123 volleyball players (63 males and 60 females; mean age, 15.8 years). The prevalence of GIRD was investigated for internal rotation differences of >10° and total rotation motion of <5°. Questionnaire items and ROM were compared between GIRD and non-GIRD patients, and sex differences in ROM were also presented. Of the participants, 38.2% (n = 47/123) had GIRD. The GIRD group showed a decrease in external rotation on the dominant side (*p* = 0.003, 1 − beta = 0.84), but this was not associated with a history of shoulder injury. Sex differences in shoulder ROM showed hypomobility in males and hypermobility in females. However, there was no association between shoulder injury and GIRD among adolescent players. There are sex differences in ROM, which should be considered in future studies.

## 1. Introduction

Volleyball is popular among people of all ages, causing fewer injuries than other sports, and it has health benefits [1,2]. However, acute musculoskeletal injuries due to overuse were reported [3,4]. These injuries occur in various body parts, including the ankle, knee, shoulder, finger, and lower back [5,6,7]. With regard to injury prevention in volleyball players, there are many reports on the ankle and knee, where the incidence of injury is high [5]. Therefore, a lack of research on the etiology and prevention of shoulder injuries was noted in a recent systematic review [5].

Notably, overuse injuries in the shoulders of volleyball players are more common than those related to the spine [8], and professional to recreational players of all ages require more attention to shoulder joint conditions. Overhead athletes, such as volleyball players, may exhibit mobility and flexibility changes in the dominant glenohumeral (GH) joint as an adaptation to their sporting activities [9,10,11,12]. This results in a much less internal rotation (IR) and more external rotation (ER) of the shoulder, and it is classified as a glenohumeral internal rotation deficit (GIRD) [10,11,12]. Many athletes develop excessive posterior capsular tightness with 90° shoulder abduction and ER ≥ 90° repetitive motion, causing the humeral head to move anteriorly and superiorly compared to the non-dominant shoulder in a throwing motion [13]. Several studies showed that these adaptations may alter posterior GH joint muscle stiffness, GH joint mobility, posterior capsule stiffness, rotator cuff ER:IR strength ratio, and joint position sense in the dominant shoulder [14,15,16,17]. Overhead athletes are more likely to develop pathologies, such as shoulder internal impingement, because of forces that resist shoulder traction, horizontal adduction, and IR during deceleration of throwing and swinging motions [18,19,20]. These factors may manifest as shoulder pain, leading to poor performance and missed practice. In sports activities, athletes often complain of pain even though they are not injured, which not only reduces their sports performance and causes them to miss practice [21,22]. Since repetitive movements, such as spiking, serving, and one-handed receiving, are inevitable for volleyball players, it is necessary to provide them with ways to care for themselves. However, most studies were conducted on baseball players, and only a limited number of studies were conducted on volleyball players alone. 

Manske et al. defined two types of GIRD: normal and pathological [23]. One is anatomical GIRD, which is a normal response in overhead athletes and has a reduced IR of <18–20° and total rotation motion (TROM) symmetry. The other is pathological GIRD, with a decrease in scapular IR motion of 18° to more than 20° and a decrease in TROM of >5° when compared bilaterally. GIRD is often present in adult volleyball players [24], but this may not be related to shoulder pain or injury, and imbalances in muscle strength around the shoulder can affect pain or injury [25]. On the other hand, Schmalzl et al. found that in adult volleyball players, being an offensive player and having a GIRD of ≥10° is associated with a higher prevalence of postero-superior impingement and decreased TROM [24]. Alqarni et al. also found that the difference in GIRD and TROM was greater in players with a history of shoulder pain compared to that in the non-pain group [26]. These two studies recommend that the GIRD criteria for defining shoulder pain in volleyball players should be an IR difference of ≥10° [24,26]. Thus, the relationship between shoulder pain and GIRD in volleyball players has not reached a certain point of view, and further study is needed. The previous studies have often focused only on spikers with high shoulder impact [9,24], and the characteristics of the shoulder joint range of motion in the court position are not clear. In addition, only few studies focused on adolescent players or sex differences [9,27,28]. The likelihood of injury increases in adolescent female volleyball players as their years of experience and level of competition increase [6]. For this reason, investigating the characteristics of shoulder range of motion (ROM) in adolescent athletes whose musculoskeletal system is still immature and taking corresponding measures may reduce subsequent shoulder pain.

On the other hand, the epidemiology of injuries from team sports differs between males and females [29]. While world-class volleyball players showed no sex differences in injury rates or location [30], this is unclear in adolescent volleyball players. The physical demands of volleyball may differ because of sex differences in the number of jumps made during a match and play style [31,32]. Hadzic et al. also reported an abnormal shoulder rotator strength ratio in male compared to female volleyball players [33]. Despite this, there are only few reports on sex differences in ROM in adolescent volleyball players. If these differences in ROM exist, there could be differences in prevention programs for shoulder disorders. Therefore, the main purpose of this study was to investigate the prevalence of GIRD with decreased TROM, whether GIRD is related to shoulder pain and court position, and sex differences in ROM among 15–17-year-old high school volleyball players. In this study, we hypothesized that (1) GIRD is present in approximately 40% of high school volleyball players [9], but is not associated with pain as it is in adult players due to the low volume of practice; (2) the prevalence of GIRD is higher in spikers [9]; and (3) female players have more joint flexibility [34].

## 2. Materials and Methods

### 2.1. Design and Ethical Considerations

This was a case–control study following the strengthening the reporting of observational studies in epidemiology checklist. This study was approved by the Ethics Committee of the Faculty of Health and Medical Care, Saitama Medical University, Japan (M-73; certified date, 19 May 2017), and was conducted in accordance with the principles of the Declaration of Helsinki. A consent of the teams, players, and their parents was obtained prior to study commencement. The study’s purpose was explained via written and verbal communication to the school principal and volleyball club coach of each high school, after which the principal’s written consent was obtained. A similar explanation was also provided to the participants and their parents, after which a written consent was obtained.

### 2.2. Participants

The inclusion criteria were male and female volleyball players aged 15–17 years belonging to the top 32 of 243 schools (male: 99 teams; female: 144 teams) included in the Saitama Prefecture High School Volleyball League, Japan, in order to homogenize the players’ competition level. This was conducted to standardize the skill level of the athletes and the intensity and frequency of their practice, thereby reducing the impact of training program on the effectiveness of the intervention.

We invited 16–32 elite high school volleyball teams across Saitama, Japan, to participate in this study from June 2017, and each team received a detailed information regarding the study, including an overview and inclusion and exclusion criteria. The exclusion criteria were players who (1) had a history of surgery in the spine, upper limbs, or lower limbs; (2) had absences from practice due to some injuries; (3) had a history of GH dislocation; (4) had structural abnormalities in the shoulder and thoracic regions, such as scoliosis and kyphotic postures; and (5) had abdominal pain or abdominal discomfort with shoulder pain [35].

Based on recent studies on GIRD in volleyball players [23,26,36,37], it was defined as a difference in IR ROM of ≥10° and a corresponding loss of TROM of ≥5° when compared bilaterally. Since this study aimed to estimate the association between GIRD and shoulder pain or court position in high school players, the case group consisted of players who currently had GIRD. The control group was selected from a group of high school players with no GIRD. To clarify sex differences in ROM, male and female groups were also made. Control groups were matched to cases based on age, sport, and number of training hours per week. One control group was selected per case group.

### 2.3. Sample Size

Sample size was calculated using power analysis application G*Power 3.1.9.7 (http://www.gpower.hhu.de/; accessed on 17 March 2020). To compare questionnaire items and physical function items between the GIRD and asymptomatic groups and between the male and female groups, the effect size d was set to 0.5 (α = 0.05, 1 − β = 0.8) for independent t-test, and the effect size w was set to 0.3 (α = 0.05, 1 − β = 0.8) for chi-square test. The level of effect size was medium for both analyses. As a result, the number of participants required were 128 cases (64 per group) for t-test and 88 for χ^2^ test. Therefore, we tried to recruit at least 128 participants at the beginning of this study. If the number of participants was lower than planned, post hoc power analysis was conducted. 

### 2.4. Data Collection

Data, including those from questionnaires and physical function tests, were collected from high school athletes. The investigation period was from July to October 2017. The survey items were demographic details, environmental factors, presence of current low back pain (LBP) during volleyball, and physical function. 

Demographic details included sex, age (years), height (cm), body weight (kg), dominant hand, and years of experience as a volleyball player. In addition, the players’ BMI (kg/m^2^) was calculated. Hand dominance was determined by the arm used to spike or serve. Participants replied to the presence or absence of pain around the joints (shoulder, elbow, knee, ankle, finger, or lumbar) during volleyball practice within one year that did not lead to a physician's visit or significantly interfere with their play. In the present survey, the presence or absence of at least one injury per year was considered. Lumbar pain was defined as pain or discomfort in the lower back within the area between the lowest ribs and buttocks [38]. Participants with symptoms associated with menstruation were not classified as LBP.

The environmental factors assessed were volleyball court position (spiker [outside spiker or middle blocker] or others [setter or libero]), spike form (bow and arrow, circular, or straight), average volleyball practice time, practice days per week, and presence or absence of static stretching after playing. 

The following items were measured as physical function tests. Vertical jump (cm), spike jump (cm), and block jump (cm) values were measured for each player and were calculated by subtracting the standing reach point from the highest reach point of each jump. Each jump was performed three times, with the highest point being recorded. Active shoulder flexion (FL), ER, IR, and horizontal abduction (HAB) ROM (°, degree), as well as trunk rotation ROM, were measured bilaterally using a plastic goniometer (GS-100; OG Wellness Inc., Okayama, Japan) at 5° increments. Spike form was checked on each player by one of the certified volleyball coaches (Y.M.) of the national sports association with more than 10 years of experience. Shoulder FL was assessed while the participant was in a supine position. Shoulder ER and IR were assessed with the participants in supine and their arms abducted at 90° in the plane of the scapula, with the scapula stabilized anteriorly at the coracoid process [12,23,39]. TROM was calculated by summing the IR and ER ROMs of each limb. GIRD measurements were calculated from the difference in IR ROM between the dominant and non-dominant shoulders. Shoulder HAB was assessed with the shoulder at 90° abduction and elbow at 90° flexion while the participant was in a prone position without trunk rotation. For trunk rotation, the participants were instructed to be in a sitting position with their feet on the floor and knees and hips at 90° flexion. The goniometer was positioned with the axis fixed in the transverse plane at the level of T1–T2, following the measurement recommendations [40,41]. The subjects were instructed to kneel against the wall to reduce the contribution of the lower body on spine rotation. Additionally, to reduce measurement errors as much as possible, all physical function tests were administered by two physical therapists (N.S. and H.N.) who were blinded to the study after being instructed on the measurement methods by another skilled physical therapist (Y.M.) with more than 10 years of experience. All ROM measurements from three trials were averaged for data analysis [37].

### 2.5. Outcomes

Primary outcomes included the prevalence of GIRD in high school volleyball players and the association between its presence and court position or pain in other parts. Secondary outcomes were sex differences in questionnaire items and ROM of the shoulder and trunk.

### 2.6. Statistical Analysis

For statistical analyses, a simple tabulation was performed for questionnaire items. For continuous variables, means were calculated with standard deviations. Comparisons between groups for primary and secondary outcomes were made using independent t-test or Mann–Whitney U-test for continuous variables and chi-squared test for categorical data. All statistical analyses were conducted using IBM SPSS Statistics for Windows, Version 25.0 (Armonk, NY, USA: IBM Corp Released 2017), and the significance level was set at *p* = 0.05.

## 3. Results

The investigation period lasted from July to November 2017, during which a convenience sample of 63 male and 60 female volleyball players (age: 15.8 ± 0.7 years) was recruited from eight public high schools. This study achieved a 100% questionnaire response rate (Table 1). There were no missing measurements for all items. The highest frequency of injury during volleyball in the previous year was in the lumbar spine, followed by those in the ankle, finger, knee, shoulder, and elbow. The percentages of spike form types were bow and arrow, straight, and circular, in that order. Stretching after practice was performed 74% (n = 91/123) of the time, but the type and duration of stretching varied. In particular, stretching time was less than 30 s in all cases (n = 91/91).

### 3.1. Primary Outcomes

#### 3.1.1. Participants’ Shoulder and Trunk ROM

The outcomes of shoulder and trunk ROM of all participants are presented in Table 2. Comparing the dominant and non-dominant side, the dominant side had more ER ROM (*p* < 0.001, 1 − β = 0.99) and less IR (*p* < 0.001, 1 − β = 0.99) and HAB ROM (*p* = 0.001, 1 − β = 0.89). Of the participants, 68.3% (n = 84/123) had an IR difference of ≥10°, and 38.2% (n = 47/123) had GIRD (IR difference of ≥10° and TROM deficit of ≥5°).

#### 3.1.2. Comparison of Questionnaire and ROM between the GIRD and Non-GIRD Group

The outcomes of the comparison between the GIRD and non-GIRD groups are presented in Table 3. This comparison showed a decrease in ER on the dominant hand side (*p* = 0.003, 1 − β = 0.84) in addition to a decrease in IR and TROM (*p* < 0.05) in the GIRD group. No relationship was found between GIRD and history of shoulder injury. No other significant differences were found in court position (*p* = 0.37), spike form (*p* = 0.90), or practice time (weekday, *p* = 0.60; holiday, *p* = 0.53) or frequency (*p* = 0.99) depending on the presence or absence of GIRD.

### 3.2. Secondary Outcomes

#### Sex Differences in Questionnaire Items and Shoulder and Trunk ROM

The questionnaire items and sex differences in ROM are shown in Table 4. Males had less years of volleyball experience and had a higher jumping ability (*p* < 0.05). The female group had longer practice times on weekdays and holidays (*p* < 0.05 and <0.001), but tended not to perform stretching after practice (*p* < 0.001). This group also had a higher percentage of non-spikers and straight arm swingers (*p* < 0.05 or 1 − β > 0.8). For injuries within one year during volleyball, the female group had a higher prevalence of ankle and shoulder injuries than the male group (*p* < 0.05 or 1 − β > 0.8).

Shoulder ER ROM showed that both groups had over 90° of hyper-rotation, with the female group having a wider ROM (male, 107.9 ± 13.1°; female, 118.1 ± 12.0°). Shoulder IR ROM showed of <90° in both groups, with a narrower ROM in the male group (male, 56.1 ± 13.6°; female, 65.7 ± 10.8°). The TROM of the female group was > 180° and had a wider range (dominant, 183.8 ± 17.6°; non-dominant, 181.3 ± 17.5°), while that of the male group was < 180° (dominant, 163.3 ± 18.0°; non-dominant, 163.0 ± 15.7°). Shoulder HAB ROM was wider in males, but both groups had a narrower range on the dominant hand side (dominant/non-dominant; male, 57.3 ± 17.1°/65.8 ± 16.8°; female, 50.8 ± 13.6°/55.3 ± 13.0°).

## 4. Discussion

Based on questionnaire and shoulder and trunk ROM measurements for adolescent volleyball players, this study investigated the prevalence of GIRD, characteristics of the GIRD group, and sex differences in ROM. Additionally, the history of injuries in the previous year was investigated. The resulting prevalence of GIRD in adolescent volleyball players was 38.2%. The GIRD group showed a decreased ER ROM, which was not related to shoulder injury, court position, years of experience, or practice time. Sex differences in ROM were wider in the female group for ER, IR, and TROM of the shoulder joint, while it was wider in the male group for HAB of the shoulder joint. Furthermore, spikers in the female group were more likely to use a straight arm swing than those in the male group. The history of injury in the previous year was similar to the frequency reported in previous studies [5,6,8], although LBP was the most frequently injured.

Overall, the participants showed excessive ER ROM and IR ROM reductions on the dominant side, similar to a previous study on adolescent volleyball players [9]. Several studies reported that GIRD occurs in overhead athletes and is the most common indication seen in the GH joint with excessive ER ROM and decreased TROM [12,23,25,42,43,44,45,46]. In this study, 38.2% had GIRD, which was similar to a study of Harput et al. where 38.5% of adolescent volleyball players had GIRD with decreased TROM [9]. The present study also considered whether court position was associated with GIRD, but no differences were found. The reason for this may be that most high school volleyball players do not have their positions clearly defined by their coaches during practice, and all players tend to spend most of their time practicing overall volleyball skills that are not specific to their court positions. This opinion seems reasonable given that 67.5% of the players were not main members of the team in the previous year. In general, the spiker's overall body load is likely to be greater in a match than in practice, so future studies may need to evaluate the shoulder condition immediately after a load equivalent to that of a match.

The TROM of all subjects was similar bilaterally and appeared to be adapted by reduced IR ROM and excessive ER ROM on the dominant side (approximately 10° each). These results suggest that the adaptation of the dominant shoulder joint occurs in adolescent volleyball players and baseball and tennis players [47,48]. On the other hand, the GIRD group showed a decrease in ER ROM and TROM on the dominant side. Kibler et al. introduced the concept of external rotation deficiency (ERD), which is defined as a difference of <5° between the ER of the throwing shoulder and non-throwing shoulder [49]. The authors propose that in baseball players, pitchers with a <5° side to side difference in ER place a greater stress on static GH stabilizers, which may result in an increased risk of injury throughout the player’s career [49,50]. In the GIRD group, an ERD of >5° on the dominant side was observed; although it was not related to the previous year's shoulder injury, it may be associated with a potential risk. These findings suggest that players with GIRD may develop pain as they continue to practice volleyball in the future. Seminati et al. reported a higher incidence of shoulder overuse than back injuries and suggested that coaches and athletes should work together to minimize the anatomic stress on the shoulder while maintaining their performance [8]. On the other hand, about 65% of players who experienced LBP did not consult their coaches or doctors [7]. Therefore, injury-related education for coaches and players may be necessary to protect adolescent volleyball players from frequent injuries. In the presence of GIRD with decreased TROM, it may be necessary to evaluate the ER ROM and then decide on an individual basis whether ER ROM or IR ROM expansion is needed.

Sex differences in ROM showed a trend toward hypomobility in males and hypermobility in females in ER and IR ROM. We could not find any studies examining the sex differences in shoulder ROM of volleyball players, but there were reports for swimmers. Mise et al. reported that shoulder joint hypomobility in males and hypermobility and the amount of practice in females were risk factors for developing shoulder disorders in young competitive swimmers (mean age, 14 years). In this study, the practice time per practice session was longer in the female group. The stabilization of the GH joint during acceleration, deceleration, and follow-through phases of spiking motion is provided by the rotator cuff muscles, which act eccentrically to compress the humeral head [51]. Hence, to prevent the development of shoulder pain, consideration of practice time may be necessary in addition to stabilization exercises for shoulder hypermobility. Actually, it is recommended to focus on improving eccentric muscle strength and correcting imbalances between the internal and external rotator muscles of adolescent female volleyball players [52]. In the male group, if the IR limitation was due to posterior capsule stiffness, a modified sleeper stretch has been shown to be effective [53]. Overall, <30 s per type of stretch for the entire body, including the shoulders, may indicate an inadequate cool-down time and carry over the risk to the next day. This short stretching time seems to reflect the desire of coaches and athletes to do more competitive practice in a limited amount of time. Generally, a static stretching time of at least 30 s is recommended after practice [54,55]; for the participants in this study (especially the male group), it may be necessary to modify the stretching method and time. Thus, stretching instructions for injury prevention may be effective to increase the shoulder joint mobility of male athletes, but this may be counterproductive for female athletes with hypermobility.

Furthermore, a higher percentage of the female group used a straight arm swing. The basic purpose of spiking in volleyball is to hit hard, and as in baseball and tennis, the angular velocity of shoulder IR is as high as 4000–7000°/s. In the maximum voluntary isometric contraction of the rotator cuff activity, it is also equivalent to 54–71% in serve and spike in volleyball compared to baseball (49–99%) [56,57,58,59]. On top of these required functional activities, Seminati et al. reported that during spiking, the circular swing has 10° less flexion angle, 15° more HAB angle, higher axial rotation speed, and 5% higher ball speed than the bow and arrow swing [60]. In addition, the association between shoulder flexion and shoulder pain during volleyball suggests the use of a circular swing, which is used by about 40% of professional indoor players [61,62]. On the other hand, the percentage of circular swing use drops to 13.3% in beach volleyball, where the jump force is lower than in indoors [63]. These reasons may explain the slightly higher percentage of circular swings in the male group with higher jumping ability and higher percentage of straight swings in the female group with lower jumping ability. It is also possible that an adaptation to expand the HAB ROM may be occurring in the male group to achieve a stronger spike. Since there were reports of a decreased HAB ROM being associated with LBP in the previous year [64], evaluating the trunk and shoulders may be necessary when an athlete is observed to be compensating for GH joint movement by over-rotating the trunk. However, it remains difficult to compare the relevance found in this study to the existing literature, as there is no set consensus on whether or not different types of spiking movements cause any adaptation in shoulder ROM. 

This study had several limitations. First, the results cannot be generalized to all volleyball players because only high school volleyball players were observed in this study, recruiting teams of the same competition level and age to homogenize the functional aspects. However, we consider that the selection of participants in this study was appropriate because the number of disorders increases with the gradual increase in the amount of practice from this age group [65,66]. Future studies are needed to longitudinally observe how adolescent volleyball players are susceptible to injuries as their skeletons mature and as they are exposed to practice for a longer period of time. Second, the accuracy of these data could not be determined because we relied on the participants’ honesty to report the presence of past injuries. Finally, in addition to GIRD with a decreased TROM, shoulder IR/ER muscle strength and shoulder joint position sense are known to be involved in shoulder joint disorders in volleyball players [25,26,33,67,68,69], but these were not considered in this study. Research on these factors in adolescent volleyball players remains lacking. However, since this study revealed sex differences in ROM and spiking motion, future studies should consider these for detailed longitudinal observations and preventive measures to prevent shoulder pain and injuries.

## 5. Conclusions

The results showed a 38.2% prevalence of GIRD with a decreased TROM in adolescent volleyball players; the GIRD group had a concomitant ERD, which was not related to sex, body composition, history of shoulder injury, years of experience in volleyball, practice time, or court position. There were sex differences in shoulder ROM, particularly in ER and IR ROM, with hypomobility in males and hypermobility in females. Females tended to have a straight arm swing more often. Thus, screening adolescent athletes regularly and designing prevention programs to address dangerous adaptive changes may be important to prevent potential shoulder injuries.

## Figures and Tables

**Table 1:** Basic attributes and environmental factors of the participants (n = 123).

Variable		
Demographic Details	Sex, n; Male/Female	63/60
	Age (years)	15.8 ± 0.7
	BMI (kg/m^2^)	20.4 ± 1.6
	Hand dominance, n; R/L	113/10
	Experience as a volleyball player (years)	3.5 ± 2.0
	Vertical jump (cm)	50.8 ± 10.6
	Spike jump (cm)	56.2 ± 12.3
	Block jump (cm)	41.9 ± 9.0
	Injury history in the previous year during volleyball, presence, n (%)	Shoulder: 11 (8.9%), Elbow:6 (4.9%) Knee: 24 (19.5%), Ankle: 51 (41.5%) Finger: 45 (36.6%), LBP: 59 (48.0%)
Environmental factors	Court position, n; spikers/others	83/40
	Spike form †, n (%); bow and arrow/circular/straight	56 (67.5)/11 (13.3)/16 (19.3)
	Main players who participated in the previous tournament, n (%)	40 (32.5), non-main player 83 (67.5)
	Practice time/day (h); weekday/holiday	3.0 ± 0.6/3.8 ± 0.5
	Number of practice/week	6.1 ± 0.9
	Static stretching after practice, n (%)	Presence: 91 (74.0%)
	Time per type of stretching after practice, n (%)	<30 s: 91 (100), ≥30 s: 0 (0%)

Mean ± SD; BMI, body mass; R, right, L, left; LBP, low back pain; spikers, outside spiker and middle blocker, others, setter and libero. † Only spikers (n = 83).

**Table 2:** Participants' shoulder and trunk ROM (n = 123).

	Variables	Dominant	Non-Dominant	*p*-Value	Power (1 − β)
Shoulder ROM	FL (degree)	178.2 ± 5.1	178.9 ± 3.5	0.17	0.24
	Asymmetry of FL (degree)	1.0 ± 3.5			
	ER (degree)	112.9 ± 13.5	102.5 ± 10.2	<0.001 *	**0.99**
	Asymmetry of ER (degree)	11.7 ± 8.6			
	IR (degree)	60.4 ± 13.6	69.4 ± 13.8	**<0.001**	**0.99**
	Asymmetry of IR (degree)	12.5 ± 9.8			
	IR difference of ≥10°, n (%)	84 (68.3)			
	TROM (degree)	173.3 ± 20.5	171.9 ± 18.9	0.59	0.08
	GIRD, n (%)	47 (38.2)			
	HAB (degree)	54.2 ± 15.8	60.7 ± 15.9	**0.001**	**0.89**
	Asymmetry of HAB (degree)	9.9 ± 9.2			
Trunk ROM	Rotation (degree)	66.3 ± 13.2	67.4 ± 12.2	0.59	0.10
	Asymmetry of rotation (degree)	1.4 ± 8.6			

Mean ± SD; ROM, range of motion; FL, flexion; ER, external rotation; IR, internal rotation; TROM, total rotation motion; GIRD, glenohumeral internal rotation deficit; HAB, horizontal abduction. Analysis carried out using independent *t*-test. * The bold values indicate statistical significance of *p* < 0.05, or a power of ≥ 0.8.

**Table 3:** Comparison of questionnaire and ROM between the GIRD and non-GIRD group.

Variables	GIRD (n = 47)	Non-GIRD (n = 76)	*p*-Value	Power (1 − β)
Sex, n (%); male/female	27 (57.4)/20 (42.6)	36 (47.4)/40 (52.6)	0.28	0.37
Age (years)	15.7 ± 0.7	15.9 ± 0.7	0.10	0.32
BMI (kg/m^2^)	20.4 ± 1.6	20.5 ± 1.6	0.57	0.06
Hand dominance, n; R/L	41 (87.2)/6 (12.8)	72 (94.7)/4 (5.3)	0.18	0.45
Experience as a volleyball player (years)	3.7 ± 2.4	3.4 ± 1.7	0.99	0.12
Vertical jump (cm)	50.2 ± 9.2	48.2 ± 10.6	0.34	0.18
Spike jump (cm)	54.9 ± 11.1	53.1 ± 12.9	0.35	0.12
Block jump (cm)	42.3 ± 9.3	40.3 ± 9.6	0.26	0.20
Injury history in the previous year during volleyball, presence				
Shoulder, n (%)	Presence: 4 (8.5)	Presence: 7 (9.2)	0.58	0.05
Elbow, n (%)	Presence: 2 (4.3)	Presence: 4 (5.3)	0.58	0.06
Knee, n (%)	Presence: 12 (25.5)	Presence: 12 (15.8)	0.16	0.44
Ankle, n (%)	Presence: 18 (38.3)	Presence: 33 (43.4)	0.58	0.12
LBP, n (%)	Presence: 21 (44.7)	Presence: 38 (50.0)	0.57	0.12
Court position, n (%); spikers/others	34 (72.3)/13 (27.7)	49 (64.5)/27 (35.5)	0.37	0.27
Spike form †, n (%);				
bow and arrow/circular/straight	22 (64.7)/5 (14.7)/7 (20.6)	34 (69.4)/6 (12.2)/9 (18.4)	0.90	0.10
Main players who participated in the previous tournament, n (%)	12 (25.5)/35 (74.5)	28 (36.8)/48 (63.2)	0.19	0.58
Practice time/day (h) weekday	3.1 ± 0.5	2.9 ± 0.6	0.60	0.47
holiday	3.8 ± 0.5	3.8 ± 0.5	0.53	0.08
Number of practice/week	6.1 ± 1.0	6.1 ± 0.9	0.99	0.05
Static stretching after practice, n (%)	Presence: 34 (72.3)	Presence: 57 (75.0)	0.74	0.07
Shoulder ROM FL (degree) dominant side	177.6 ± 6.7	178.6 ± 3.8	0.71	0.16
non-dominant side	178.5 ± 0.9	179.4 ± 2.6	0.51	0.14
ER (degree) dominant side	108.3 ± 13.0	115.7 ± 13.1	0.003 *	**0.84**
non-dominant side	103.5 ± 10.7	101.8 ± 9.9	0.551	0.14
IR (degree) dominant side	56.1 ± 13.6	63.0 ± 12.9	**0.004**	0.78
non-dominant side	73.8 ± 14.2	66.7 ± 12.9	**0.02**	0.78
TROM (degree) dominant side	164.4 ± 19.8	178.9 ± 19.1	**0.001**	**0.97**
non-dominant side	177.3 ± 18.3	168.6 ± 18.6	**0.015**	0.69
HAB (degree) dominant side	54.7 ± 16.0	53.8 ± 15.7	0.59	0.06
non-dominant side	62.5 ± 15.8	59.5 ± 16.0	0.27	0.17
Trunk ROM Rotation (degree) dominant side	66.8 ± 13.5	66.0 ± 13.2	0.80	0.06
non-dominant side	68.4 ± 13.8	66.8 ± 11.2	0.25	0.10

Mean ± SD; ROM, range of motion; GIRD, glenohumeral internal rotation deficit; BMI, body mass index; LBP, low back pain; spikers, outside spiker and middle blocker; others, setter and libero; FL, flexion; ER, external rotation; IR, internal rotation; TROM, total rotation motion; HAB, horizontal abduction. † Only spikers (GIRD, n = 34; non-GIRD, n = 49). Analysis carried out using Mann–Whitney U-test. * Bold values indicate a statistical significance at *p* < 0.05 or a power of ≥ 0.8.

**Table 4:** Sex differences in questionnaire items and shoulder and trunk ROM.

Variables	Male (n = 63)	Female (n = 60)	*p*-Value	Power (1 − β)
Age (years)	15.7 ± 0.7	15.9 ± 0.7	0.03 **	0.34
BMI (kg/m^2^)	20.2 ± 1.5	20.7 ± 1.7	0.20	0.39
Hand dominance, n; R/L	56 (88.9)/7 (11.1)	57 (95.0)/3 (5.0)	0.18	0.34
Experience as a volleyball player (years)	2.9 ± 1.9	4.2 ± 2.0	**<0.001**	**0.95**
Vertical jump (cm)	56.4 ± 7.1	41.2 ± 6.1	**<0.001**	**1.00**
Spike jump (cm)	63.0 ± 8.6	44.1 ± 7.3	**<0.001**	**1.00**
Block jump (cm)	48.0 ± 6.6	33.9 ± 6.0	**<0.001**	**1.00**
Injury history in the previous year during volleyball, presence				
Shoulder, n (%)	Presence: 3 (4.8)	Presence: 8 (13.3)	0.10	**0.94**
Elbow, n (%)	Presence: 4 (6.3)	Presence: 2 (3.3)	0.36	0.12
Knee, n (%)	Presence: 11 (17.5)	Presence: 13 (21.7)	0.56	0.13
Ankle, n (%)	Presence: 21 (33.3)	Presence: 30 (50.0)	**0.06**	0.88
LBP, n (%)	Presence: 35 (55.6)	Presence: 29 (48.3)	0.42	0.20
Court position, n (%); spikers/others	49 (77.8)/14 (22.2)	34 (56.7)/26 (43.3)	**0.01**	**0.99**
Spike form †, n (%);				
bow and arrow/circular/straight	37 (75.5)/8 (16.3)/4 (8.2) *	19 (55.9)/3 (8.8)/12 (35.3) *	**0.008**	**1.00**
Main players who participated in the previous tournament, n (%)	14 (22.2)	26 (43.3)	**0.01**	0.51
Practice time/day (h) weekday	2.8 ± 0.5	3.1 ± 0.5	**0.03**	**0.90**
holiday	3.5 ± 0.5	4.1 ± 0.2	**<0.001**	**1.00**
Number of practice/week	6.1 ± 0.9	6.1 ± 1.0	0.91	0.05
Static stretching after practice, n (%)	Presence: 55 (87.3%)	Presence: 36 (60.0%)	**<0.001**	**1.00**
Shoulder ROM				
FL (degree) dominant side	177.6 ± 5.8	178.8 ± 4.2	0.22	0.25
non-dominant side	178.7 ± 4.2	179.3 ± 2.6	0.43	0.15
ER (degree) dominant side	107.9 ± 13.1	118.1 ± 12.0	**<0.001**	**0.99**
non-dominant side	99.0 ± 8.9	106.2 ± 10.3	**<0.001**	**0.98**
IR (degree) dominant side	55.3 ± 14.1	65.7 ± 10.8	**<0.001**	**0.99**
non-dominant side	64.0 ± 12.5	75.1 ± 12.9	**<0.001**	**0.99**
TROM (degree) dominant side	163.3 ± 18.0	183.8 ± 17.6	**<0.001**	**0.99**
non-dominant side	163.0 ± 15.7	181.3 ± 17.5	**<0.001**	**0.99**
HAB (degree) dominant side	57.3 ± 17.1	50.8 ± 13.6	**0.02**	**0.62**
non-dominant side	65.8 ± 16.8	55.3 ± 13.0	**<0.001**	**0.96**
Trunk ROM				
Rotation (degree) dominant side	64.8 ± 14.5	67.8 ± 11.7	0.28	0.23
non-dominant side	65.6 ± 12.7	69.4 ± 11.5	0.09	0.39

Mean ± SD; ROM, range of motion; GIRD, glenohumeral internal rotation deficit; BMI, body mass index; LBP, low back pain; spikers, outside spiker and middle blocker, others, setter and libero; FL, flexion; ER, external rotation; IR, internal rotation; TROM, total rotation motion; HAB, horizontal abduction. † Only spikers (male, n = 49; female, n = 34). Analysis carried out using Mann–Whitney U-test. * *p* < 0.05. Analysis was carried out using z-test and Bonferroni-test. ** Bold values indicate a statistical significance at *p* < 0.05 or a power of ≥0.8.

## Data Availability

The data presented in this study are available on request from the corresponding author.
